# Dissecting membrane interfacial cellular processes: an in vitro reconstitution approach

**DOI:** 10.1140/epjs/s11734-024-01108-3

**Published:** 2024-02-16

**Authors:** Ayush Panda, Amaresh Kumar Mahakud, Amit Beniwal, Barsha Rani Pradhan, Mohammed Saleem

**Affiliations:** 1 School of Biological Sciences, National Institute of Science Education and Research, Bhubaneswar, India; 2 Homi Bhabha National Institute, Mumbai, India

## Abstract

Cellular membrane interfacial processes are highly complex phenomena that are challenging to explore in vivo, often limiting their mechanistic understanding. The plethora of participating biological entities and their unprecedented complex interactions might also mask their core guiding principles. In vitro reconstitution experiments with a reductionist bottom-up approach attempt to dissect the biological complexity by recreating biological reactions in their simplest form. This review focuses on the cellular membrane interfacial processes crucial for cellular transport, neurodegeneration, and host–pathogen interactions. We discuss how reconstitution methodologies have played a crucial role in addressing many of the fundamental questions dealing with the mechanisms of clathrin-dependent or independent endocytosis and the Endosomal Sorting Complex Required for Transport (ESCRT) pathway. In addition, we have discussed how reconstitution-based approaches helped explore membrane-mediated alterations in the onset of neurodegenerative diseases and host–pathogen interactions. Importantly, the reconstitution approach, though predominantly applied but not limited to addressing questions on cellular membrane shaping, is gradually gaining attention across multiple areas of cell biology.

## Introduction

In the 1920s, the idea to create a self-organizing reconstituted system was initially investigated in the realm of chemical systems. In the last few years, this has been constructively employed to analyze self-assembly-related phenomena in cell biology [53]. The best illustration of this is the in vitro reconstitution of cytoskeletal filaments and their communications with motors and other binding proteins*,* which allowed the analysis of their lively behavior outside the cell [59]. Mimicking complicated cellular processes, like cell division and motility-like events, requires their restriction to minimal spaces inside a bilayer membrane. Such studies, comprised of purified proteins and cell-free extracts offer a complementary method to live-cell experiments, making both systems crucial to understanding the diverse biological processes. These kinds of minimal cell-free approaches give three significant ideas. First, it helps building molecular models of various cellular processes, independent of complex cellular environments; second, the simplicity of reconstitution systems governed by only physical laws allows for the discovery of unexpected behaviors; and third, being compliant with various mathematical models helps take into account numerous variables like space, time and others to illustrate the biological processes [65]. Modern-day cell biologists have adopted in vitro reconstitution for various biochemical processes, further than cytoskeletal processes. The exploration of membrane trafficking studies was initiated through the discovery and isolation of the Sec mutant in yeast, which blocked membrane traffic [101]. Further, through reconstitution with the help of purified proteins, CopI and CopII were discovered to partake in protein transport. It also helped in better understanding of various cellular processes like protein translocation in endoplasmic reticulum and enzyme performances [35, 45]. Researchers have also explored the impact of membrane parameters on various cellular processes in cell-free mediums. Through reconstitution, researchers have discussed the regulation of integral membrane proteins with respect to the neighboring lipid nanodomains [64]. For example, a membrane with an unvarying thickness that depends explicitly on local constituent lipids may lead to the clustering of misfit integral membrane proteins to lessen membrane deformation [51]. The impact of membrane force on integral proteins can be studied through reconstitution. One such report explains the function of mechanosensitive channels such as MscL and MscS related to the force applied to the local membrane environment [22, 91]. The reconstitution approach also finds its way into the cellular trafficking processes, including the involvement of the membrane environment. By employing synthetically produced liposomes and purified Rab GTPase proteins, the inherent ability of these proteins to tether to well-defined membrane bilayers has been discovered. Further investigation from these reconstitution studies favors the unique working model wherein Rab-family small GTPases operate as a real membrane tether for facilitating membrane tethering affairs in eukaryotic membrane trafficking [78]. There are two major membrane models in reconstitution systems, i.e., supported-lipid bilayers (SLBs) and lipid vesicles of various sizes, which are utilized to investigate various cellular processes. Formation of planar SLBs can be achieved by bursting or flattening small lipid vesicles on glass surfaces. These bilayer surfaces have been utilized for lipid diffusion studies and broadly for biomedical investigations. [98]. Giant unilamellar vesicles (GUVs) are made of a lipid bilayer and are typically around 1–100 microns in size. With this model, studies like lipid diffusion in the plane, and morphological alterations out of the plane like protrusions or invaginations, can be performed [66, 115]. GUVs can easily be observed under an optical microscope, and they are widely considered to be the best mimicking models for the cell plasma membrane (PM) [14]. They are particularly useful in visualizing the assembly of proteins engaged in membrane deformation, fusion, and fission [111]. Small Unilamellar Vesicles (SUVs) and Large Unilamellar Vesicles (LUVs) range in size from tens to hundreds of nanometers, respectively, and are widely used in protein-binding assays. These unilamellar vesicles, used in conjunction with electron microscopy, can give important insights into the effects of proteins on membrane shape [111].

Reconstitution studies of the recent past have had a substantial influence on the current understanding of the numerous complicated cellular processes like regulation of membrane trafficking, cytoskeletal processes, and protein transport. This review delves into the utilization of this minimalistic cell-free reconstitution approach to investigate cellular processes like clathrin-dependent and independent endocytosis, and the Endosomal Sorting Complex Required for Transport (ESCRT) pathway. Also, reconstitution studies of membrane involvement in neurodegeneration and pathogen-mediated cellular alterations have been described.

## Clathrin-mediated endocytosis

**Fig. 1:**
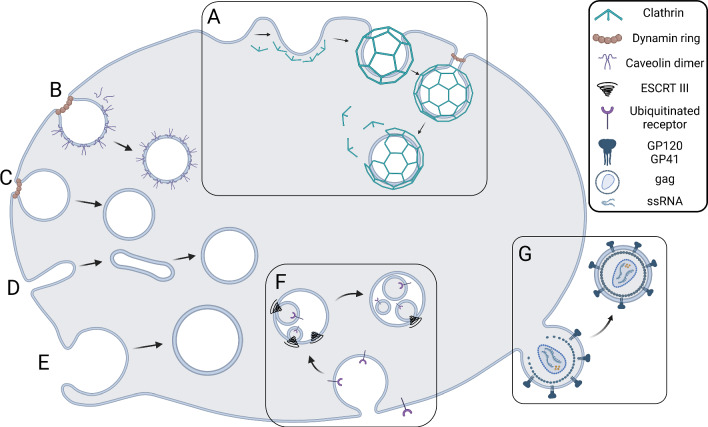
A schematic representation of membrane linked processes in case of clathrin-dependent (**A**) and independent endocytosis (**B**–**E**), ESCRT pathway (**F**), and host–pathogen interactions (**G**)

Endocytosis is the process of internalization of diverse cargo molecules via small membrane vesicles that transport them from the cell surface into the cytoplasm [52]. Clathrin-mediated endocytosis (Fig. 1A) is one of the major pathways of endocytosis, which has been widely studied over the past few decades. Reconstitution experiments have played a pivotal role in advancing the understanding of clathrin-mediated endocytosis, by elucidating the specific functions of individual proteins involved in the process and enabling the characterization of the associated physical parameters. By offering a bottom-up approach, reconstitution experiments have helped delineate the minimum machinery sufficient to carry out each step of this process in which a planar membrane undergoes gross deformation to finally become a spherical bud [23].

### Initiation of endocytosis and cargo recruitment

**Fig. 2:**
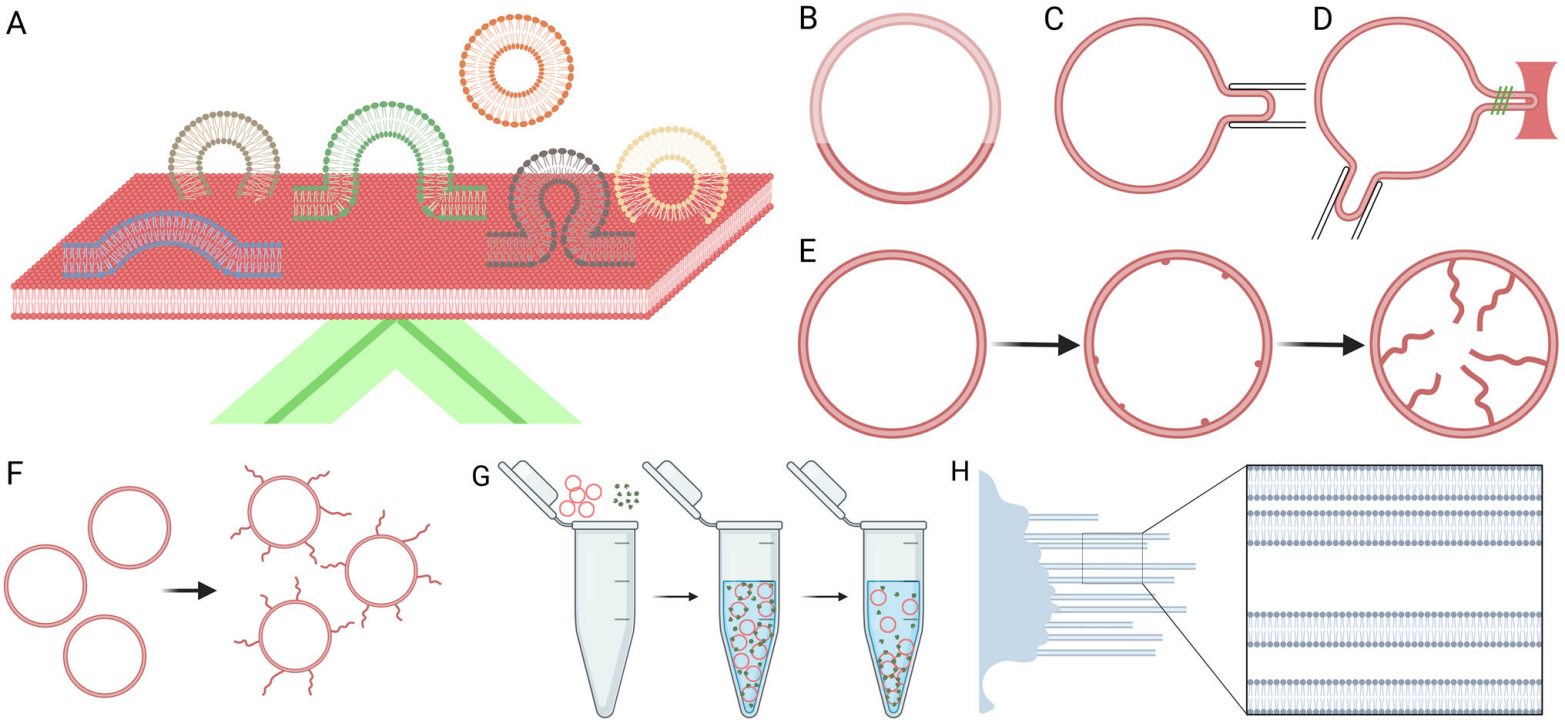
Schematic of membrane-associated reconstitution methods, **A** Formation and visualization of supported lipid bilayers (SLBs) through TIRF microscopy, **B** Phase-separated GUVs, **C** Micropipette aspiration, **D** Optical tweezer with micropipette aspiration, **E** Membrane tubular invaginations, **F** Membrane tubulation, **G** Lipid co-sedimentation assay, **H** SMrt: supported membrane tubes

Endocytosis is initiated at the PM by the nucleation of the ‘pioneer module’ proteins, which primarily include FCHo 1/2 (F-BAR proteins), Eps15 and intersectin [52]. FCHo1/2 proteins exhibit specific binding to the PM and engage scaffold proteins such as Eps15 and intersectin, which further leads to the recruitment of the downstream adaptor protein, AP2. It has been suggested that FCHo1/2 proteins play a major role in sculpting the initial bud site and facilitating clathrin machinery recruitment for clathrin-coated vesicle (CCV) formation [43]. These initiation events have been demonstrated via reconstitution experiments, some of which are described here. The predominant targeting of these proteins to the PM has been demonstrated through a lipid co-sedimentation assay conducted by Henne et al., which revealed the preferential binding of F-BAR proteins to reconstituted liposomes enriched with phosphatidylinositol 4,5-bisphosphate [PI(4,5)P2], a defining marker of the PM (Fig. 2G). Furthermore, F-BAR proteins were shown to generate tubulations of increasing curvatures in PI(4,5)P2 liposomes, with the curvature being proportional to the protein concentration (Fig. 2F). Further, studies from Day et al. complemented these findings by illustrating that liquid-like assemblies, formed through weak multivalent interactions between Eps15 and FCHo1, promote the recruitment of downstream adaptors, thereby facilitating the rapid maturation of the endocytic vesicle. Using reconstituted PI(4,5)P2-containing giant unilamellar vesicles (GUVs), incubated with fluorescently labeled Eps15 and FCHo1, they have neatly demonstrated the formation of partitioned protein-rich domains on the membrane surface, caused by interactions between Eps15 and FCHo (Fig. 2B). Eps15 or FCHo1 individually, or FCHo1 with truncated Eps15, inhibited the formation of these partitioned domains on the GUVs. This was further recapitulated on flat lipid multi-bilayer stacks, where FCHo1 and Eps15 synergistically co-assembled to form membrane-associated condensates [27, 102, 122]. Reportedly, FCHo proteins contain an intrinsically disordered linker domain that binds and activates AP2 [46, 116]. The AP2 adaptor protein is composed of four major subunits (α, β2, σ2, and μ2) and bridges the clathrin scaffold to PI(4,5)P2 in the membrane and the transmembrane protein cargo. In the cytosol, it is in its ‘closed’ conformation in which its cargo binding sites are blocked by the β2 subunit. When it is recruited to the endocytic site, it undergoes a large conformational change [48]. Quantitative mass spectrometry studies have previously revealed that while there is a substantial level of AP2 within CCVs, the quantity of FCHo2 proteins is scant [11]. This result agrees with the suggestion that Eps15 and FCHo are pushed to the periphery of the developing pit, and are ultimately left behind in the PM when the vesicle buds out. Zaccai et al. support this idea by showing that the binding of PI(4,5)P2-containing membrane to AP2 competes off the FCHo linker domain [128]. They performed the liposome pull-down assay where the addition of PI(4,5)P2-containing liposomes displaced the linker from preformed AP2core·FCHo linker complexes, into the soluble fraction. Further, by employing cryo-electron tomography they have illustrated the activating conformational changes in AP2 which occur upon binding to liposomes. Partlow et al., in a study published around the same time as Zaccai et al., described a similar mechanism of AP2 activation and emphasized the importance of membrane in the process using a variety of reconstitution experiments. They described a new conformation of AP2 before cargo recruitment, called ‘primed’ which occurs in the presence of PI(4,5)P2-containing SLBs and FCHo2 [85]. SLBs are planar lipid membranes formed on a solid support, which provide advantage over liposomes by avoiding the high curvature and tension. By visualizing SLBs via confocal microscopy, the authors showed the clustering of cargo peptides with tyrosine-based recognition motifs, caused by the interaction of AP2 and FCHo in a membrane environment. Cargo binding by the ‘primed’ membrane-associated AP2 complex induces further conformational changes, ultimately leading to the canonical ‘open’ conformation, in which the clathrin binding site is revealed. This initiation of endocytosis is followed by two major membrane processes i.e. membrane bending and scission, which are discussed below.

### Membrane bending

As cargo gets recruited to the endocytic site by AP2, clathrin also gets engaged and starts to polymerize into a lattice around the developing bud. One model, proposed by Kelly and coworkers, suggests that AP2 is sufficient to drive the formation of clathrin-coated buds in vitro [55]. They observed that numerous clathrin-coated buds were formed when AP2-loaded and cargo-supplemented PI(4,5)P2 liposomes were incubated with clathrin. Another model suggests that curvature-inducing adaptor proteins bend the membrane at the periphery of the developing bud and this curvature is stabilized by the oligomerization of clathrin into a cage-like structure around the bud [71]. One such well-studied membrane curvature-inducing protein is epsin1, which contains a tightly-folded, membrane-binding domain called ‘ENTH’ at its N-terminus [112]. This ENTH domain preferentially binds to PI(4,5)P2 through its N-terminal unstructured amino acid stretch, which assumes an amphipathic helix structure upon binding to the membrane [31]. The mechanism behind the bending of the membrane by epsin1 is mainly explained by two models. An early model proposed that shallow insertion of the amphipathic helix into the outer leaflet of the membrane, pushing the head groups apart would cause membrane deformation, thereby reducing the energy required to curve the membrane. This model was based on observations that monomeric epsin1 and its ENTH domain caused high tubulation and even fragmentation in PI(4,5)P2-containing total brain lipid liposomes. They also observed the recruitment and polymerization of clathrin on lipid monolayers mediated by epsin1 [31]. The other model is based on EPR assays on reconstituted POPC/POPS/PI(4,5)P2 vesicles to study the membrane docking topology of the ENTH domain, which revealed a unique slanted orientation of its penetrating amphipathic helix [127]. The authors argue that the curvature caused by the epsin1 ENTH domain mainly derives from its slanted penetration into the membrane and its self-association on the membrane surface into a protein aggregate, as opposed to being monomers in the previous model. Membrane tension inhibits membrane deformation during clathrin-mediated endocytosis by hindering the curvature of flat membranes into dome-like structures. In fact, multiple studies show that low membrane tension causes the clustering of PI(4,5)P2, thereby explaining how mechanically favorable regions are selected as the endocytic site [93]. Saleem et al. reconstituted the recruitment and polymerization of clathrin at varying membrane tensions in GUVs, by changing the osmotic conditions. While clathrin coats caused extensive budding under low membrane tension, high tension fully inhibited its polymerization. Yet, they observed that membrane deformations occurred under unfavorable membrane tension conditions when the GUVs were incubated with clathrin in the presence of epsin [99]. A recent study further underlined the role of epsin, by showing that a GUV-based system, developed to generate vesicles in vitro, required the presence of epsin1 for the formation of clathrin-coated pits (CCP), which are the precursors of vesicles [15]. They further showed, contrary to previous reports, that AP2 was dispensable to this process of forming CCPs.

### Membrane fission

In clathrin-mediated endocytosis, the scission of vesicles from the membrane is highly regulated by the Dynamin family of GTPases [57]. The role of dynamin in membrane fission (also called as scission) has been demonstrated in many reconstitution experiments in vitro [26, 103]. In one of the early studies proposing a GTP-dependent constriction model, purified dynamin was incubated with phosphatidylserine liposomes, and through transmission electron microscopy (TEM), the binding of dynamin to liposomes was observed [114]. Dynamin bound to liposomes and formed tubes of diameters comparable to that of the neck of clathrin-coated invaginations. GTP treatment of these tubes caused structural changes and constriction, forming vesicles observed by both electron microscopy and dynamic light scattering. A different model, based on experiments involving real-time light microscopy of reconstituted membranes, proposed that it is the GTP-dependent ‘twisting’ of dynamin that leads to membrane fission [97]. A reconstitution system was developed by drying a drop of lipid mixture on a coverslip, which was then mounted on a glass slide to create a microchamber. The lipids were then reorganized into stacks of flat membrane bilayers by the addition of aqueous buffers. When purified dynamin was injected into this reconstituted system, tubes were formed which underwent supercoiling upon subsequent GTP addition. The twisting motion was further confirmed by the rotation of streptavidin-coated polystyrene beads attached to the biotinylated dynamin scaffold on these tubules, upon the addition of GTP. Membrane fission catalyzed by dynamin has also been reconstituted on a flat substrate called the ‘SUPER’ (SUPported bilayers with Excess Reservoir) template [81, 88, 89]. These templates are prepared by depositing a single lipid bilayer on 5-μm silica beads, by liposome fusion on the surface of the bead in a high salt concentration solution such that a ∼2.4-fold excess reservoir is present. When dynamin was added to this system in the absence of nucleotides, it led to the formation of dynamin-coated long tubules. Vesiculation was observed from the template in the constant presence of GTP, which was further confirmed by a sedimentation assay showing the presence of small fluorescent vesicles in the supernatant. SUPER templates have since been used to study the fission activity of dynamin mutants in centronuclear myopathy [19], regulation of dynamin2 by EHD ATPase [49], the role of PHD conformational switch in dynamin-catalyzed fission [110], Bin1-mediated inhibition of dynamin [60] and to pull-down dynamin-binding nanobodies [32]. Reportedly, Dar and coworkers developed a high-throughput in vitro assay system consisting of a flow cell containing an array of membrane tubes resting on a passive surface, which allowed the real-time analysis of membrane fission reactions by fluorescence microscopy [24]. This in vitro system of supported membrane tubes, named SMrt, was engineered to have the dimensions of the neck of a clathrin-coated pit (Fig. 2H). They observed that an intact dynamin helical scaffold progressively constricted the underlying membrane tube, upon GTP hydrolysis, until it reached the critical radius of 7.3 nm. At this critical dimension, the tube underwent scission, accompanied by the splitting of the scaffold. In a subsequent study, Dar et al. employed SUPER templates and SMrt to show that the membrane-binding pleckstrin-homology domain (PHD) of dynamin was dispensable to the scission process [25]. Dynamin was re-engineered to replace its PHD with a polyhistidine linker, but it still constricted and severed SMrTs. Here, the polyhistidine linker of the re-engineered dynamin helped to recruit it to the NTA-containing membranes. This helped to mimic the main function of the PHD, i.e. membrane insertion, which has been previously shown to be critical for dynamin-catalyzed vesicle scission [90]. Recruitment of dynamin to the neck region of the developing clathrin-coated pit is mediated by BIN/amphiphysin/Rvs (BAR) domain-containing proteins, which bind to the C-terminal proline-rich domain (PRD) of dynamin via their SRC homology 3 (SH3) domains [36, 37]. BAR proteins are also involved in sculpting the membrane in the neck region of the developing bud, which can form a template for dynamin oligomerization. It has been shown by optical trap experiments that at low concentrations, dynamin nucleation only occurred on high-curved tubes, pulled from a GUV using optical tweezers [96]. This indicates that spontaneous polymerization of dynamin can be triggered at the neck of closing CCP in vivo. Meinecke and coworkers reported the cooperative nature of the recruitment of dynamin and BAR proteins, endophilin and amphiphysin [72]. They observed that dynamin recruitment to GUVs was highly accelerated in the presence of endophilin and amphiphysin. The addition of endophilin caused dynamin to bind more efficiently and led to the clustering of both proteins on the membrane. Upon changing the order of protein addition, endophilin did not independently bind to the GUV but got readily bound to the membrane within seconds of dynamin addition. When dynamin, amphiphysin or endophilin, were added together with GTP, vesicle release from the GUVs was observed. This vesicle release was dependent on the interaction between the BAR proteins and dynamin, as dynamin mutants without the PRD could not trigger vesiculation in the GUVs. These results were complemented by Neumann et al. who reported that Dynamin2-catalyzed vesicle release from SUPER templates was promoted by the addition of isolated N-BAR domain of endophilin and full-length amphiphysin [82]. Snead and coworkers added further insight by underlining the fact that BAR domain-containing proteins usually also contain bulky intrinsically disordered regions that cause crowding on the membrane surface, thereby destabilizing lipid tubules and driving their fission [107]. They employed negative stain TEM to observe that full-length amphiphysin divided 200-nm vesicles into high-curved vesicles with an average diameter of 22 nm, as compared to the N-BAR domain which only transformed the vesicles into long tubules. After scission from the membrane, the clathrin coat soon undergoes disassembly to uncoat the vesicle, which is then trafficked to its target site for fusion. Single-particle fluorescence experiments with high time resolution have been employed to monitor the disassembly of the clathrin coat driven by the chaperone Hsc70 in an in vitro system [9, 111].

While clathrin-mediated endocytosis remains the most widely studied pathway for membrane trafficking at the PM, various other pathways, in which clathrin is inessential, have emerged that transport cargo from the cell surface to its interior. Reconstitution experiments have helped in providing a mechanistic insight into this wide variety of pathways, that has been described in the subsequent section under the umbrella of clathrin-independent endocytosis.

## Clathrin-independent endocytosis

Several pathways for clathrin-independent endocytosis (CIE)(Fig. 1B-E) have been elucidated over the past few decades which perform important functions in membrane trafficking [69] and through reconstitution linked methods more information has been dug out. Small GTPases like RhoA, Rac, and Cdc42, in addition to caveolae (Fig. 1B) and macropinocytosis(Fig. 1E), have all been implicated in CIE pathways [100]. BAR (Bin/Amphiphysin/Rvs)-proteins have also been shown to participate in CIE, by helping create invaginations in the membrane that aid vesiculation, in some cases independent of dynamin [42]. Actin and actin-associated proteins have been reported to be major players in these processes. Shiga toxin, produced by *Shigella dysenteriae* is a well-studied bacterial toxin that manages to efficiently enter cells, even when clathrin is inhibited [63]. It has been shown to bind with glycosphingolipid Gb3 in the membrane and undergo CIE to enter the cell. Cytosol-free GUVs containing 5% Gb3 were incubated with fluorophore-labeled B-subunit of Shiga toxin (STxB) and observed by fluorescence microscopy [94]. When membrane tension was decreased by increasing the osmotic pressure of the bath, STxB-induced lipid reorganization causing negative membrane curvature and the formation of inward membrane tubular invaginations (Fig. 2E). In a subsequent report, the authors further elucidated the role of actin in promoting dynamin-independent scission of these inward membrane invaginations [95]. 5% Gb3 GUVs with and without 30% cholesterol were incubated with STxB and then shifted to 4 °C which triggered membrane scission, but only in the GUVs containing cholesterol. In addition, they generated a model membrane system that incorporated the actin machinery—actin, the Arp2/3 complex, VVCA-His, ADF/cofilin, and gelsolin. This machinery was encapsulated into liposomes by the inverted emulsion technique. Actin polymerization was triggered by the diffusion of salts, thereby forming cellular actin cortex-mimicking ‘actin shell’ at the inner leaflet. This cortical actin shell led to the cholesterol-dependent but dynamin-independent scission of the inward invaginations induced by STxB. Endophilin-A2 also participates in this process by stabilizing these membrane invaginations [92]. Liposomes with asymmetric bilayer composition were prepared by inverse emulsion. In the reverse of the actual cellular situation, STxB was encapsulated inside liposomes followed by the external addition of endoA2 to the medium, which triggered the straightening of the coiled invaginations. To further understand this morphological transformation induced by endoA2, the authors performed dynamic measurements by using optical tweezers to pull membrane nanotube tethers from GUVs held by a micropipette (Fig. 2D). This allowed them to study the force exerted by protein binding on the tether and control the elongation velocity and radii of the nanotubes. Upon injection of endoA2, the tube retraction force fell to zero, suggesting that endoA2 forms an external scaffold on the membrane of the tether, stabilizing them and preventing their spontaneous scission. Finally, the authors proposed a model where actin, endophilin-A2, and dynamin additively contribute to the scission in CIE (Fig. 1C). The binding of STxB to lipids also induces membrane reorganization [121] and reordering to form long-range lipid orders, as shown by experiments involving atomic force microscopy of 1% Gb3 lipid films on a mica surface [108]. In a biological membrane, the fatty acid chain of Gb3 varies significantly in saturation and α-hydroxylation, which might modulate the CIE of the toxin. Supported membranes were doped with Gb3s of varying fatty acid chains which led to significant change in the phase behavior of the membrane and consequently modulated the binding of Shiga toxin [80]. A major clathrin- and dynamin-independent pathway is the CLIC/GEEC pathway (Fig. 1D). The fusion of PM-derived primary tubulovesicular carriers, called clathrin-independent carriers (CLICs) results in the formation of endocytic structures, containing endocytosed glycosylphosphatidylinositol-anchored proteins (GPI-APs), called GEECs (GPI-AP enriched early endosomal compartments) [70]. The glycosphingolipid (GSL)-dependent biogenesis of CLICs is triggered by a carbohydrate-binding protein, galectin-3 (Gal3), which has been implicated in the CIE of CD44 and β1-integrin [61]. The authors prepared GUVs with a plasma-membrane-like lipid composition, including a 5% GSL mixture. Gal3 did not readily bind to the GUV membrane, in agreement with the fact that it needs glycosylated proteins for its membrane recruitment. To mimic the binding of Gal3 to cargo proteins on the membrane, Gal3 was His-tagged and forced to bind NTA-containing GUVs. This resulted in the formation of tubular membrane invaginations, underlining Gal3’s membrane bending ability.

Transitioning from these investigations describing the endocytic events happening at the PM, our focus now shifts to another crucial cellular pathway, ESCRT, in which reconstitution experiments have helped unveil additional layers of complexity in the dynamic orchestration of cellular membrane dynamics.

## Endosomal sorting complex required for transport

The endosomal sorting complex required for transport (ESCRT) pathway (Fig. 1F) has been shown to play key roles in various membrane-remodeling events. In opposition to the previously well-characterized dynamin-catalyzed scission which occurs on the exterior of the bud neck, ESCRT possesses the unique ability to catalyze scission from within the membrane neck [104]. The biogenesis of multivesicular bodies (MVBs) is mediated by this ESCRT system, which involves the sorting of ubiquitinated cargo coupled with the scission of intraluminal vesicles (ILVs) [38]. Four major multimeric protein complexes have been reported in mammalian and yeast cells: ESCRT-0, ESCRT-I, ESCRT-II, and ESCRT-III along with an ATPase Vps4. In yeast, ESCRT-III is composed of Vps20, Snf7, Vps24, and Vps2, together with the ATPase Vps4 which triggers their disassembly. Multiple reconstitution models, including GUVs [2, 8, 87, 106], SLBs [18, 76, 87], and LUVs (Large unilamellar vesicles) [8, 44, 47, 79, 83] have been employed over the years to elucidate how various proteins participate in the ESCRT pathway. Wollert et al. reconstituted this pathway by adding purified recombinant yeast ESCRT-III proteins to cellular membrane mimicking GUVs [7, 124]. The addition of three components of ESCRT-III (Vps20, Snf7 and Vps24) sequentially was sufficient for the formation of ILVs which were completely detached from the surrounding membrane. Subsequently, the addition of the other two components, Vps2 which recruits Vps4 and Vps4 itself, led to the formation of a second wave of ILVs, which were identified by their internal GFP content from the surrounding medium. In the subsequent report, Wollert et al. reconstituted the biogenesis of MVBs in GUVs and defined the roles played by ESCRT-0, -I, -II and -III complexes in the internalization of membrane-tethered fluorescent ubiquitin (model cargo) via ILVs [123]. ESCRT-0 was shown to cluster ubiquitin, as shown by their strong colocalization in domains on the GUV membrane. Synergistic application of both ESCRT-I and -II induced buds into the lumen of the GUV, but the proteins themselves localized to the bud neck. ESCRT-I and -II further confined cargo into these buds, which was demonstrated by the lack of fluorescence recovery after photobleaching the cargo in the buds. Finally, ESCRT-III was recruited by ESCRT-I and -II to the membrane neck and carried out scission of ILVs. Pfitzner et al. collated many years of research and employed multiple reconstitution experiments to propose a multistep membrane-remodeling pathway that includes a sequential polymerization of ESCRT-III subunits, combined with a preferential sequence of Vps4-mediated disassembly reactions [86]. They employed a similar reconstitution set-up as Chiaruttini et al. [18], in which SLBs were obtained by bursting GUVs on cleaned glass coverslips, which were built into a flow chamber. After flushing Snf7 into the chamber which formed patches on the SLB, they incubated labeled Did2 or Ist1 and varying combinations of ESCRT-III subunits at equimolar concentrations on the same. Consequently, they established that cascade-like recruitment is observed for the ESCRT subunits. More recently, membrane tension has been reported to play a regulatory role in the recruitment of the ESCRT-III complex. Mercier and colleagues used GUV as model membranes to study the recruitment of purified CHMP4B, the human Snf7 homologue, under different tension conditions, which were modulated by two means—changing the osmolarity of the surrounding medium and by aspirating GUVs with micropipettes at different pressures (Fig. 2C). They noted that the rate of CHMP4B polymerization was inversely related to the membrane tension [73]. Similar results were obtained by Booth et al. who were working yeast ESCRT-III proteins. They established that there exists a negative feedback mechanism from membrane tension that regulates ESCRT-III remodeling activity [10]. ESCRT proteins have also been reported to induce lipid phase separation in vitro, which aids in membrane budding [12]. Total internal reflection fluorescence microscopy (TIRFM) of SLBs (Fig. 2A) revealed that ESCRT-II underwent cholesterol-dependent self-assembly to form clusters of 10–100 molecules. These clusters formed ordered domains in the underlying membrane and excluded a liquid-disordered phase-specific dye. A recent study showed similar results for *Entamoeba histolytica* ESCRT-III proteins incubated with GUVs made of ternary lipid mixtures, either homogeneous in phase or exhibiting coexistence of both liquid-disordered and -ordered phases [3, 5]. ESCRT-III induced domain formation in the model membranes and triggered the generation of ILVs from the liquid-ordered phases. A major limitation of these experiments involving sequential addition of ESCRT proteins to reconstituted membranes, is that previously added proteins remain in the system throughout the experiment, which makes it difficult to assign a specific task to each protein. Avalos-Padilla et al. circumvented these issues by developing a microfluidic technology to trap GUVs which allowed them to monitor the ESCRT-mediated remodeling by each protein as they could wash away the unbound proteins before adding the next [4]. These experiments revealed the ability of ESCRT proteins to regulate bud size and generate ILVs by modulating the membrane stiffness and spontaneous membrane curvature. Reports suggest that ESCRT is also involved during infection by certain parasites like, *Plasmodium falciparum* [3, 5]. *P. falciparum* recruits its ESCRT machinery to increase EV production from red blood cells, which have lost their own vesicular network. The authors reconstituted the formation of EVs by MVBs and membrane shedding, in GUVs using the purified recombinant proteins. Injection of ESCRT-III Plasmodium proteins in GUVs caused outward budding. ESCRT also participates in the process of phagocytosis in Entamoeba histolytica, which is responsible for amoebiasis in humans. Using GUVs, mutant trophozoites, and recombinant proteins (rEhVps20, rEhVps32, rEhVps24, and rEhVps2), Avalos-Padilla et al. revealed that sequential protein assembly led to the formation of ILVs [6].

ESCRT proteins also play an important role in cell division. Homologs of ESCRT-III proteins in *Sulfolobus acidocaldarius* mediate cell division, and show functional analogy to Z ring present in *E. coli* [41]. Labelled and purified cell-division proteins (Vps4, ESCRT-III, and CdvA) were co-reconstituted in GUVs, which led to specific membrane deformation. CdvA is a membrane-binding cytoskeletal protein which acts a recruitment platform for the ESCRT-III machinery. Reconstitution of such cytoskeletal proteins and the cytoskeleton itself into GUVs has been a fascinating challenge in synthetic biology, to define the minimal machinery needed for artificial cell division. This was achieved by Merkle et al. who reported the formation of an actin-based cytoskeleton within GUVs [75]. The Schwille group has also reconstituted the Min system in vitro, which plays an important role in the positioning of the Z ring for division in *E. coli* [130]. More recently, they employed lipid vesicles to demonstrate the full reconstitution of the divisome machinery, and observed the condensation of FtsZ into a ring and its subsequent constriction [58]. Apart from ESCRT, FtsZ ring and protein crowding, which have all been reported to induce fission in giant vesicles, membrane-bound His-tagged fluorescent proteins have also been shown to carry out division. His-tagged GFP was added to the exterior of GUVs, which consequently caused spontaneous curvature, followed by constriction and fission of the vesicles [113].

Having discussed the pivotal role of reconstitution experiments in deciphering natural cellular processes happening at the membrane interface, our focus now shifts towards exploring their instrumental contribution in unraveling the involvement of membranes in disease pathogenesis. Specifically, we delve into their role in comprehending conditions such as neurodegeneration and the complex interactions between hosts and viruses.

## Membrane linked alteration in case of neurodegenerative diseases

The significance of membrane interfaces in promoting neurodegenerative disorders such as Alzheimer’s and Parkinson’s disease has become increasingly prominent over the past few decades. The interaction between amyloidogenic proteins associated with neurodegenerative diseases and the neuronal membrane implicated in the onset and progression of neurodegeneration has been studied extensively through various reconstitution methods [66]. Amyloid Beta (Aβ), an intrinsically disordered protein that is known as a causative agent of Alzheimer’s Disease (AD), has been reported to undergo fibrillation in close association with membrane deformation [68]. Studies involving tapping mode atomic force microscopy have previously demonstrated the interplay between planar lipid bilayers, made by the fusion of lipid vesicles on mica surfaces, and soluble Aβ monomers [126]. They reported that acidic lipids and the transmembrane domain of Aβ were essential to fibrillogenesis. Subsequently, experiments involving a combination of TIRFM and AFM showed that Aβ aggregation occurred exclusively on the gel phase DPPC domains of DOPC/DPPC supported bilayers [20]. These DPPC domains being platforms for the concentration and aggregation of the peptides supports the idea that lipid rafts are involved in Aβ aggregation in vivo. The interaction of cholesterol with Aβ aids in its oligomerisation and promotes pore formation in cholesterol-rich membrane domains. The enriched binding of Aβ with the cholesterol-rich membrane domains is because of the altered biophysical characteristics, including the width of the bilayer, membrane coat hydrophobicity, curvature, and lipid dynamics [28]. The density of lipid packing defect in the lipid bilayers seems essential in initiating the nucleation and amplifying hydrophobic interactions of Aβ on the myelin membrane model’s surface, which shows the extent of deformation [115].

Neurofibrillary Tangles observed in Alzheimer’s disease are localized in the neuronal cytoplasm and consist of aggregated tau protein [39]. Barré and Eliezer demonstrate that the repeat region of tau, known for mediating the tau microtubules interactions, also plays a pivotal role in constituting the proteolysis-resistant core within the ordered structure of the disease-associated tau aggregates associated with lipid micelles and vesicles. Alterations in lipid compositions, membrane curvature and membrane fluidity, and protein concentration variations significantly impact the membrane-tau interaction and the membrane disruption kinetics. Some in vitro studies have demonstrated that tau-linked membrane disruption involves the extraction of lipids from the bilayer, forming rigid scaffolds composed of both peptides and lipids. The formation of rigid peptide-lipid complexes accelerates the fibrillation of tau when incubated with liposomes [29]. Tau's surface activity guides its interaction with the membrane, stimulating the formation of β-sheet oligomers through misfolding and self-assembly. These oligomers trigger the growth of tau fibrils, ultimately contributing to their assembly in diseased tissue [67]. Although membrane–tau interaction and secretion of tau involve phosphorylation and oligomerisation, it is susceptible to membrane composition changes, particularly cholesterol and sphingomyelin content [74]. Tau interacts with the negatively charged vesicles or vesicles, mimicking the lipid composition of the neuronal plasma membranes and forming well-ordered, rigid, and toxic phospholipid complexes [1]. The accumulation of rigid peptide–lipid complexes could be correlated with intercellular transmission since it is known that the cellular internalisation of tau aggregates is considered to induce the intracellular fibrillation of the tau [56]. Monolayer studies confirmed that tau preferentially interacts with negatively charged membranes at the air–water interface, this interaction disrupts the conformation of the lipid membranes by unsettling the lipid packing at the molecular level and causing a complete disturbance in the morphological integrity of lipid bilayers [50]. The hallmark of Parkinson’s Disease is the presence of abnormally structured Lewy bodies comprising aggregated α-synuclein—a presynaptic protein of unknown function [109]. The binding of α-Syn on pipette-aspirated GUVs increases the membrane area, depending on the lipid composition, membrane tension and vesicle size [105]. Higher membrane fluidity enhances the binding of α-Syn to membrane vesicles, which is reported to induce membrane curvature [33]. Monomeric α-Syn forms lipid bilayer tubes and cylindrical micelles upon interacting with the membrane vesicles [13]. From the pathological perspective, oligomeric and fibrillar synuclein are reported to be more potent [62]. For α-Syn to effectively bind to the membrane vesicles, a minimum of 20% of the membrane’s constituent lipids must be negatively charged [119]. Liposomes rich in cholesterol, especially the ones resembling the synaptic vesicles, bind more efficiently to α-Syn oligomers [118].

## Host–pathogen interaction

Insight into the interactions between viruses and cellular membranes has been garnered by researchers through multiple reconstitution experiments, in conjunction with an array of biophysical and structural techniques [14]. These studies have played a pivotal role in deepening our understanding of the intricate molecular mechanisms that underlie crucial processes in the viral life cycle, encompassing viral entry, assembly, and other significant stages. Here, we particularly focus on the retrovirus, human immunodeficiency virus (HIV) and its membrane linked processes. The assembly and release of newly synthesized viral particles is an important step in the replication cycle of HIV, which occurs at the PM of the hijacked host T-cells (Fig. 1G). After assembly, these viral particles bud off, being packaged inside host membrane-derived lipid envelopes. The lipid composition of these viral lipid envelopes significantly differs from that of the host PM [16]. They have been reported to be disproportionately rich in sphingomyelins, glycosphingolipids, cholesterol, and PI(4,5)P2. This composition, together with the highly ordered lipid organization of viral envelopes, is strikingly similar to that of lipid raft domains in cell membranes. Multiple studies have thereby investigated how viral structural proteins interact with lipids and manage to surround themselves with such a specialized lipid environment [30]. One such viral protein is the HIV-1 group-specific antigen (Gag), which plays an important role in the assembly and budding of the HIV-1 virus particle at the PM. The N-terminal MA domain of HIV-1 Gag proteins, which undergoes co-translational modification with a myristate moiety or acyl chain, preferentially interacts with PI(4,5)P2, hence targeting it to the PM [21, 84]. Liposome flotation assays have revealed that Gag proteins without the myristoyl group failed to bind with the membrane [117]. Gui et al. developed a minimal model membrane system based on GUVs to study the various factors that play a role in the Gag-mediated budding of viral particles [40]. They showed that the budding of minivesicles from the GUV occurred only upon the addition of RNA molecules and urea, which caused the multimerization of Gag. RNA, acting as a ‘chaperone’, prevents promiscuous binding of Gag to any acidic lipid in the membrane and specifically targets Gag to PI(4,5)P2-containing domains. Liposome-based assays have been used to show that the RNA bound to the MA domain of Gag is necessary for ensuring specific binding to PI(4,5)P2. To further determine the primary driving force of Gag membrane binding, Wen and coworkers measured protein binding to GUVs, in both disordered (Ld) and ordered (Lo) phases, but of the same membrane charge, maintained by constant PS concentration [120]. This revealed that Rous sarcoma virus Gag protein is associated with the membrane, based solely on membrane charge, and independent of membrane order. Contrary to previous findings that Gag proteins are targeted to pre-existing lipid rafts, Yandrapalli et al. demonstrated that self-assembled Gag proteins caused a local reorganization to form their own specific PI(4,5)P2/Cholesterol nanoclusters in inner PM-mimicking SLBs [125]. They also report that Gag proteins are primarily partitioned into liquid-disordered regions of the inner leaflet. Complementing their work are previous studies by Keller et al., who used GUVs to show that multimerized Gag derivative proteins bound to the disordered phase of these model membranes, thereby underlining that much more complex biophysical processes are involved in membrane binding by Gag proteins [54]. The cellular ESCRT machinery is recruited to the viral assembly site and facilitates viral budding [17]. Reconstitution experiments with GUVs have successfully recapitulated this recruitment of ESCRT to sites of myristylated Gag clusters on the membrane. The authors have elucidated the ESCRT assembly cascade involving ALIX, CHMP4, CHMP3, and CHMP2, which follows the formation of Gag clusters. Apart from Gag, glycoproteins like the protruding gp120 and transmembrane gp41, which form ‘spikes’ on the viral particle, can be a factor in promoting viral envelope assembly [77]. Supported lipid bilayers were formed by the fusion of gp41-reconstituted vesicles on a mica surface, followed by the adsorption of gp120 onto the SLB from a solution. Self-assembly of wire-like structures on the SLB surface was observed by atomic force microscopy, emphasizing the importance of glycoprotein–lipid interactions in driving viral particle assembly.

## Perspective

In the dynamic realm of cell biology, researchers have been armed with an expanding arsenal of techniques over the years to unravel the intricacies of cellular life. Reconstitution experiments using purified components and controlled conditions remain a powerful approach to identify the minimal set of biochemical activities for studying membrane biology. Yet, within this powerful tool lies a crucial caveat: how do we ascertain the physiological relevance of a reconstituted system? Reconstitution experiments are reductionist in nature, and it can be challenging to determine whether a reconstituted system accurately represents the behavior of the original cellular phenomena. The answer lies not in dismissing these experiments but rather in recognizing their pivotal role as a stepping stone—a means to construct new models, reframe questions, and ultimately design experiments in living cells to validate or refute their predictions. As Stachowiak and Kirchhausen note in their narrative, the advent of newer and more advanced technology like lattice light sheet microscopy and cryo-electron microscopy tomography is promising as it offers tools to complement reconstitution experiments, but in living systems [111]. Cellular processes as described in this review are complex and highly intricate. Reconstitution experiments help to demarcate what is ‘sufficient’ to carry out a cellular process, without having to take into account the minor roles played by the intricate network of components that exist inside living systems. We believe that, as the role of membranes becomes larger and more defined in the wide landscape of cellular processes, reconstitution experiments will continue to help biologists break down and understand complex cellular systems.

In this review, our emphasis has centered on the reconstitution of interfacial processes at the cell membrane. However, due to the breadth of the subject, we acknowledge that there are numerous valuable topics we may not have covered. For a more comprehensive exploration, we encourage readers to refer to [34] on the reconstitution of cytoskeletal processes at the membrane and [129] on biological membrane organization.

## Data Availability

No data associated in the manuscript.
